# Which second line treatments after ESA failure?

**DOI:** 10.18632/oncotarget.20873

**Published:** 2017-09-14

**Authors:** Sophie Park, Charikleia Kelaidi, François Dreyfus

**Affiliations:** Sophie Park: CHU Grenoble Alpes, Clinique Universitaire d’Hématologie, GRENOBLE Cedex, FRANCE

**Keywords:** myelodysplastic syndromes, erythropoiesis stimulating agents, lenalidomide, azacitidine, outcome

In last JCO May 2017, we have published a paper entitled “Outcome of Lower-Risk Patients With Myelodysplastic Syndromes Without 5q Deletion After Failure of Erythropoiesis-Stimulating Agents” [1]. This was a large, multinational study based on registries and institutional databases and patient data from 2 GFM (Groupe Francophone des Myélodysplasies) phase II-III clinical trials of second-line treatments (azacitidine, lenalidomide [2, 3]) for patients with non-del 5q lower risk myelodysplastic syndromes having failed erythropoiesis stimulating agents (ESA). The purpose of the study was to compare the outcomes after disease-modifying second-line treatments or best supportive care (BSC).

Thirty-nine percent of patients received second-line treatment, 61% best supportive care. OS and CI incidence of AML were not different between the three groups. A time-dependent, multivariate-adjusted analysis was used. Based on results of azacitidine in higher-risk MDS patients and lenalidomide in del 5q MDS, it was anticipated that active therapy by azacitidine or lenalidomide as second-line treatments after ESA failure would be associated with improved outcomes of interest, i.e. survival and AML transformation. The results showed, on the contrary, that neither of active treatments improved outcomes. The following points may help interpret those findings.

Time-dependent analysis took into account the time elapsed between ESA-failure and second-line treatment introduction and multivariate analysis adjusted on well-known prognostic variables like age, sex and IPSS-R. However, IPSS-R was known at time failure for most patients but only for a fraction of patients at time of second-line treatment introduction. A median of one year elapsed between ESA failure and second-line treatment initiation. One can speculate that there were potentially more patients with progressive disease in the active treatment groups as compared with best supportive care. Moreover, best supportive care was a treatment by default, which was initiated at time of ESA failure. Patients may have been chosen not to receive an active treatment at ESA failure and later on because the disease burden may have been felt to be not severe enough to initiate such a treatment. Events like AML transformation may have been unnoticed in the BSC group due to less active bone marrow surveillance.

In addition, other parameters such as transfusion burden and ferritin may be important for prognosis in the second-line treatment setting.

Toxicity of treatments, which could have shed some light in the comparison of mortality between active treatments and best supportive care, was not registered in registries and institutional databases.

Very few patients were transplanted in this cohort within the given follow-up. The study probably covers a period of time where bone marrow transplantation was an option seldom implemented for those elderly patients.

The mutational burden was not assessed in either of therapeutic groups. This is important because mutations may predict response to treatments, overall survival and also because mutations dynamics may be altered by disease-modifying treatments [4].

The sequence of second-line treatments may be relevant. For example, hypomethylating agents did better than BSC in terms of survival in patients without del 5q after lenalidomide failure [5].

The study challenges the usefulness of second-line treatments for lower-risk MDS patients. Azacitidine+/-erythropoietin and lenalidomide+erythropoietin yielded transfusion-independence in 14% and 24% of patients, respectively, in the two clinical trials integrated in our study [2, 3]. Assuming that there is no response with BSC, it is probably clinically relevant to treat symptomatic lower-risk MDS patients with azacitidine or lenalidomide for quality of life. In addition, 3-year overall survival after inclusion was 70% in the clinical trial of azacitidine+/-erythropoietin, a rather favorable estimate. Moreover, a randomized trial of lenalidomide vs. placebo showed transfusion independence in 27% vs. 0, respectively, in ESA-refractory patients with lower-risk non-del 5q MDS [6]. Direct comparison of long-term outcomes with best supportive care versus azacitidine or lenalidomide in randomized controlled trials would provide the necessary evidence to make recommendations on the use of those agents in second line.

Our recent study confirmed our previous observation that duration of response to ESA <6 months, including primary failure, is an adverse prognostic factor in lower-risk MDS predicting a higher risk of AML transformation, although not reduced survival [7]. This finding adds a dynamic component of therapeutic response to the well-known pre-therapeutic prognostic values composing the IPSS-R and could help in selecting patients for bone marrow transplantation early after first-line treatment failure.

Altogether, these elements lead us to several new avenues for prospective future trials:

-the use of the duration of response to ESA<6 months as a marker of a worse prognosis in the subgroup of lower risk IPSS-R patients

-a better selection of the patients with a higher chance of response to lenalidomide and HMA (G polymorphism in the CRBN gene, DNMTA3 mutation and low baseline serum EPO level for patients who could benefit from lenalidomide therapy [3], and SF3B1 mutation for patients receiving HMA [2]),

-privilege the sequence lenalidomide and then HMA which yields better ORR and OS [5],

-try new trials aiming at reversing the natural progression of the disease as protecting the hematopoietic cells from chronic oxidative stress by low dose deferasirox (M Meunier and S Park, Oncotarget 2017, in press),

-think about new ways of immunotherapy or micro-allogeneic transplantation [8] adapted to these lower risk MDS patients, not too toxic but aiming at eradicating the mutated clones by the GVL effect or by unlocking the inhibitory immune checkpoints.
